# A Meta-Analysis of Risky Sexual Behaviour among Male Youth in Developing Countries

**DOI:** 10.1155/2015/580961

**Published:** 2015-01-29

**Authors:** Yifru Berhan, Asres Berhan

**Affiliations:** Hawassa University, P.O. Box 1560, Hawassa, Ethiopia

## Abstract

The purpose of this meta-analysis was to assess the association between risky sexual behaviour and level of education and economic status in male youth. Previous tests of the association of risky sexual behaviour with levels of education and economic status have yielded inconsistent results. Using data from 26 countries, from both within and outside Africa, we performed a meta-analysis with a specific focus on male youths' risky sexual behaviour. We applied a random effects analytic model and calculated a pooled odds ratio. Out of 19,148 males aged 15–24 years who reported having sexual intercourse in the 12 months preceding the survey, 75% engaged in higher-risk sex. The proportion of higher-risk sex among male youth aged 15–19 years was nearly 90% in 21 of the 26 countries. The pooled odds ratio showed a statistically significant association of higher-risk sex with male youth younger than 20 years, living in urban centers, well educated, and of a high economic status. The overall proportion of condom use during youths' most recent higher-risk sexual encounter was 40% and 51% among 15–19-year-olds and 20–24-year-olds, respectively. Our findings suggest that male youth's socioeconomic status is directly related to the likelihood that they practice higher-risk sex. The relationship between income and sexual behaviour should be explored further.

## 1. Introduction

Although there is no international consensus, the United nations (UN) defines youths as individuals between 15 and 24 years of age [1]. The risky sexual practices among youth may include having multiple sexual partners, early sexual debut, engaging in unprotected sexual intercourse, and engaging in sex with older partners [2–4]. However, this meta-analysis was interested in engaging in unprotected sexual intercourse with nonregular partner.

Previous reports have shown that youth's risk taking sexual behavior and their vulnerability to HIV and other sexually transmitted infections (STIs) are significantly related [5, 6]. A World Health Organization (WHO) systematic review estimated that 50% of HIV transmission occurs among youth aged 15–24 years [7], possibly because young people lack adequate life skills to protect themselves from unplanned pregnancies and STIs [8]. Risky sexual behaviors developed during youth may also influence sexual behavior in adult life, thus increasing the cumulative risk of acquiring and transmitting an STI [9–11].

Upon closer examination, the proportion of male youth with multiple sexual partners was more than four times that of their female counterparts [12]. This evidence is supported by a separate report that revealed a twofold increase in multiple sexual partners when sexually active young men and women were compared [13]. Similarly, the use of condoms by young men is substantially lower than that of adult men [8]. Unsafe penetrative heterosexual sex is thus considered the major cause of HIV transmission and propagation in many developing countries [12].

According to World Health Organization (WHO), about 85% of the world's youth live in developing countries [1]. Using the Demographic and Health Survey (DHS) and United Nations (UN) population statistics, the 2010 UNAIDS report revealed that the prevalence of multiple sexual partners among male youth aged 15–25 years has declined from 12%–14% in 1999–2003 to 8%–10% in 2004–2009. Findings regarding the association of risky sexual behaviour and educational level or wealth status are not consistent. Before HIV transmission became understood, more highly educated and wealthier individuals were reported as practicing risky sex that exposed them to HIV infection [14, 15]. Once the impact of AIDS was recognised in the 1990s, however, higher educated people were thought to be better at practicing safer sex [16, 17]. Conversely, other studies have demonstrated a significant association of unsafe sexual behaviour among people with secondary and above level of education [9, 18]. Two systematic reviews also found that higher educational attainment was associated with a greater risk of HIV infection [19, 20].

Other studies have demonstrated a relationship between risky sexual behaviour and increased economic status, suggesting that the high prevalence of risky sexual behaviour among the wealthy is likely to increase their vulnerability to HIV infection [21, 22]. Economic capability has also been found to be strongly associated with risky sexual behaviors in Cameroon [22]. However, a review that examined the association between socioeconomic status and risk of HIV infection suggested that poor individuals were not particularly at risk of HIV infection [23]. Finally, a study among university students found that predictors of risky sexual behaviour may be related to students' and their parents' socioeconomic status as well as their place of residence.

There is also evidence that university students who were educated in urban high schools are more likely to engage in risky sexual practice [24]. Similarly, teens presenting in primary care settings in urban environments seem to be at higher risk for HIV, STIs, and substance use [5].

In summary, almost all previous transnational systematic reviews have focused on Sub-Saharan Africa (SSA) and indicate that better educated and wealthier individuals are at elevated risk for HIV transmission due to their risky sexual behaviour [19–22, 24–26]. This contradicts previous assumptions that poverty constitutes the primary driver of risky sexual practice and HIV infection in SSA [10, 27]. However, it remains unclear how consistently risky sexual behaviour is associated with a higher level of education and income level across Africa and some other non-African countries.

The purpose of our meta-analysis was to assess risky sexual behavior (practicing sex with neither a spouse nor a cohabiting partner without condom) among male youth in different parts of the world with regard to age, residence, educational level, and socioeconomic status. We expected to find that male youth with relatively high socioeconomic status would report more risky sexual behaviour.

## 2. Methods

### 2.1. Data

The Demographic and Health Survey (DHS) has collected household-based data for over 20 years in developing countries. Using a cross-sectional study design with large nationally representative samples, surveys are administered in each country that employs the same measures and similar questionnaires. In the majority of these surveys, a two-stage cluster sampling design with households in urban and rural strata has been used to select study respondents. Details of the sampling and data collection methodology for the DHS are described elsewhere [28]. The data used for this meta-analysis were collected between 2003 and 2009 in 26 countries and released for public use on the MEASURE DHS website [28].

Data from 20 of the 26 countries included in this analysis were from Sub-Saharan Africa (SSA) (Benin, Burkina Faso, Cameroon, Chad, Cote d'Ivoire, Ethiopia, Ghana, Guinea, Kenya, Liberia, Malawi, Mali, Niger, Nigeria, Rwanda, Sierra Leone, Tanzania, Uganda, Zambia, and Zimbabwe). The remaining data were from Bolivia, Cambodia, Guyana, Haiti, Nepal, and Vietnam.

### 2.2. Measures

In all DHS, higher-risk sex is defined as sexual intercourse that occurred within the 12-month period prior to the survey with an individual who was neither a spouse nor a cohabiting partner. In this meta-analysis, practicing higher-risk sex and failure to use condoms during the last higher-risk sexual encounter were considered to be indicators of risky sexual behavior.

In most of the DHS, the educational levels of male youth were grouped in the following categories: no education, primary, secondary, and above. We dichotomized respondents' education level as either no or only primary education, relative to secondary education, and above. We assessed household wealth in quintiles, utilizing an index comprising such factors as water source, type of toilet facility, materials used for housing construction, ownership of various durable goods, ownership of agricultural land, ownership of domestic animals, and ownership and use of mosquito nets [28]. For this meta-analysis, we divided the wealth index into two categories, namely, lowest or low (i.e., the first or second quintile) and middle to highest (i.e., the third through fifth quintiles).

### 2.3. Analysis

By design, this study is a secondary data analysis. Using Review Manager Version 5 software [29], we determined pooled odds ratios and associated 95% confidence intervals to describe the relationship between higher-risk sex and male youth's age, residence, educational level, and wealth index. We calculated pooled odds ratios across the countries by means of the Mantel-Haenszel (M-H) statistic (the DerSimonian-Laird method or random effect model). Using the *I* ^2^ statistic [100% × (chi square − degree of freedom)/chi square] [30], we assessed the nature and extent of heterogeneity across the surveys. Because the heterogeneity among the findings in the fixed effects model across countries was significant (*I* ^2^ > 50%), we applied a random effects analytic model to account for intersurvey variation and to provide a more conservative effect than a fixed model would have provided. The *I* ^2^ statistic was used to assess the variability among the included studies and above 50% was considered as significant.

## 3. Results

Of 19,148 male youths who reported having sexual intercourse in the 12-month period prior to the survey, 75.2% practiced higher-risk sex (93% and 67% in age of 15–19 years and 20–24 years, resp.). The proportion of higher-risk sex among male youth aged 15–19 years was nearly 90% in all countries studied except Cambodia, Ethiopia, Nepal, Niger, and Vietnam.

**Figure 1:**
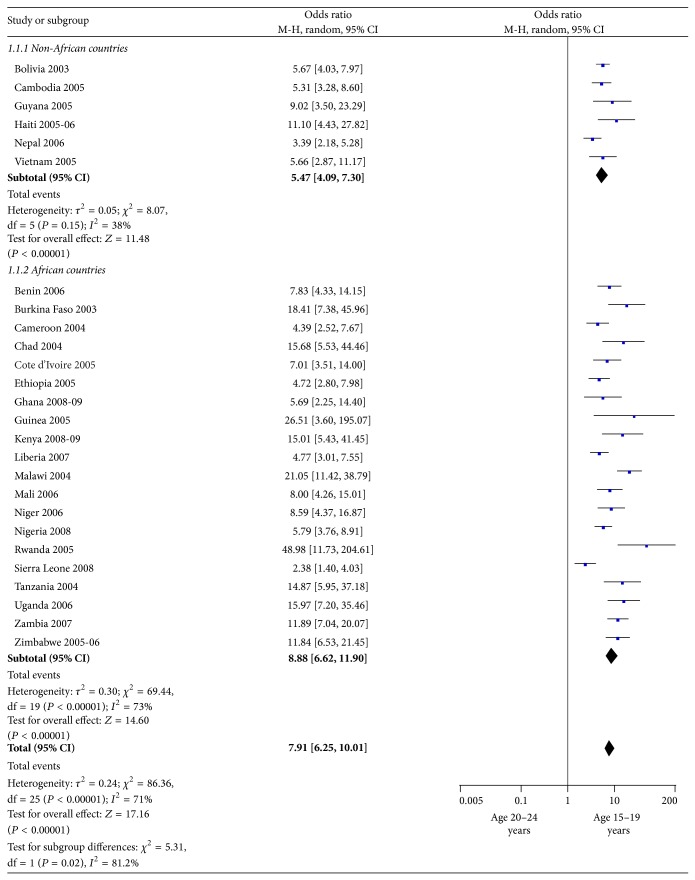
Among male youths aged 15–24 years, higher-risk sex practices in past 12 months by age group (2003–2009). M-H = Mantel-Haenszel statistic; CI = confidence interval.

The odds ratios for all countries revealed a statistically significant association of higher-risk sex with youth in the age range of 15–19 relative to 20- to 24-year-olds (Figure 1). Across countries, male youth under 20 years were about 8 times more likely than were male youth aged 20–24 years to have had higher-risk sex in the last 12 months (OR = 7.9; 95% CI: 6.25–10.01). A subgroup analysis demonstrated that the odds ratio for African male youth was higher than that for the non-Africans (OR = 8.9; 95% CI: 6.62–11.90 and OR = 5.5; 95% CI: 4.09–7.30, resp.). In other words, the age discrepancy was stronger for African male youth than for non-Africans, with younger males much more likely to report higher-risk sex.

We also examined the proportion and odds ratios of higher-risk sex among male youth in regard to their place of residence. Higher-risk sex was prevalent among male youth living in urban areas of all surveyed countries except in Cote d'Ivoire, Haiti, Tanzania, and Uganda (where higher-risk sex was not associated with area of residence). In contrast, the Kenya DHS revealed a statistically significant association of higher-risk sex among male youth living in rural areas. Across countries, male youth in urban areas were 2.6 times more likely than were rural youth to report higher-risk sex (OR = 2.6; 95% CI: 2.12–3.36) and the corresponding *I* ^2^ (85%) indicated a statistically significant heterogeneity.

**Figure 2:**
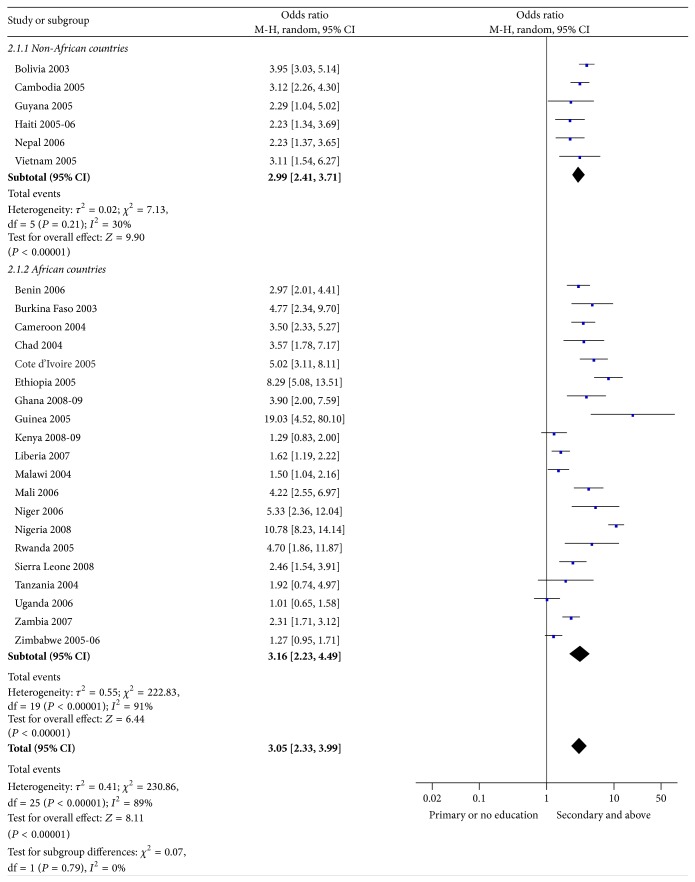
Higher-risk sex practices in past 12 months by educational attainment (2003–2009). M-H = Mantel-Haenszel statistic; CI = confidence interval.

As presented in Figure 2, in all countries but Kenya, Tanzania, Uganda, and Zimbabwe, higher-risk sex was strongly associated with youths' level of education. The pooled odds ratio also showed a statistically significant association between higher educational attainment and higher-risk sex (OR = 3.1; 95% CI: 2.33–3.99).

**Figure 3:**
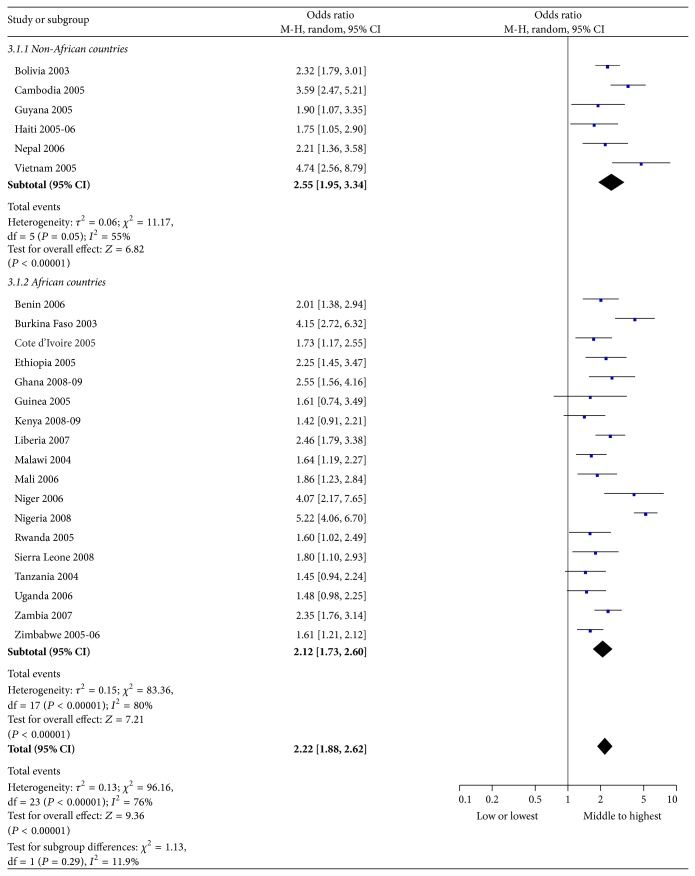
Higher-risk sex practice in prior 12 months by wealth index (2003–2009). M-H = Mantel-Haenszel statistic; CI = confidence interval. Note: we divided the wealth index into two categories, namely, lowest or low (i.e., the first or second quintile) and middle to highest (i.e., the third through fifth quintiles), to make it fit for the meta-analysis software.

Figure 3 reveals that youth who practiced higher-risk sex practice were likely to be characterized by the middle to highest wealth index in the majority of the countries studied. The total odds ratio suggests that, across countries, male youth in the middle to highest wealth index were 2.2 times more likely than low-wealth youth to report higher-risk sex (95% CI: 1.88–2.62). Although proportionally there were more higher-risk sexual practices among male youth with a higher wealth index, the odds ratios did not demonstrate a statistically significant association in Guinea, Kenya, Tanzania, and Uganda. In Burkina Faso, Cambodia, Niger, Nigeria, and Vietnam, the odds ratio was higher than in other countries.

**Figure 4:**
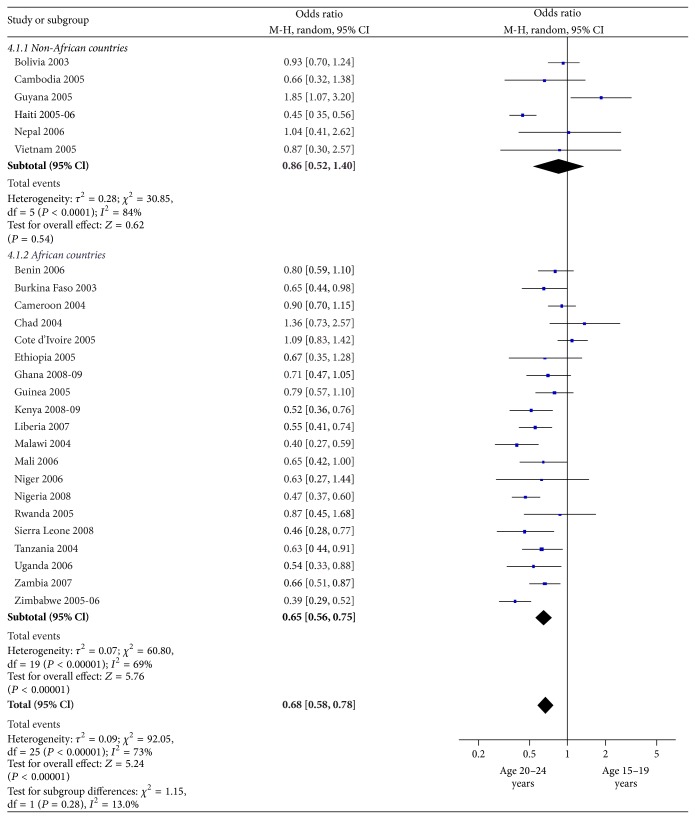
Condom use during last higher-risk sexual encounter by age group (2003–2009). M-H = Mantel-Haenszel statistic; CI = confidence interval.

We present in Figure 4 the proportion of youth using condoms during their last higher-risk sexual encounter and the pooled odds ratio in relation to youths' age groups. The overall proportion of condom use during the last higher-risk sexual encounter was 40% among 15–19-year-olds and 51% in the 20–24-year category. The pooled odds ratio revealed that youth aged 20–24 years were more likely to use condoms during their last higher-risk sexual encounter than those aged 15–19 years (OR = 0.68; 95% CI: 0.58–0.78; *I* ^2^ = 73%). In other words, the use of condom by youth aged 15–19 year was 32% less than those aged 20–24 years. In fourteen countries, however, the meta-analysis did not demonstrate a statistically significant difference between the two age categories.

## 4. Discussion

Using nationally representative multicountry data, this meta-analysis identified the most vulnerable group of male youth for STI including HIV. The strong association of higher-risk sexual behavior with higher socioeconomic status, better living conditions in terms of income, and higher educational attainment invites further research.

The issue of youth's sexual behavior and its consequences is a concern for virtually every generation and is likely to continue to be a challenge in the future [31]. Adolescents' risky sexual behavior is a public health priority because of its effects on sexuality and unplanned pregnancy during adulthood and the risk of acquiring and propagating sexually transmitted infections including HIV [21–23].

As we expected, we found that risky sexual behavior was most prevalent among youth living in urban areas who had completed secondary education and whose family income fell in the middle to the highest wealth index quintile. It remains unclear why male youth characterized by higher socioeconomic status, better living conditions, and higher educational attainment were more likely to engage in higher-risk sexual behavior and thus be particularly susceptible to HIV infection [23, 25, 26]. We raise this question because better educated people are generally thought to be more aware of the effects of risky sexual practices [5], which are expected to guide them in making decisions that reduce risk. However, regardless of geographic location, this meta-analysis demonstrates increased risk taking sexual behavior among well-educated male youth.

We are left with the question as to whether wealth or education is more important or influential in driving the link to risky sexual behavior. This area is in need of further investigation. However, we expect that the wealth status of the majority of male youth in the included countries is a reflection of that of their parents: that youth from well-to-do families are more likely to have better incomes that may contribute to experimenting with sex and engaging in sex with nonregular or noncohabitating partners. This has been suggested by other studies [8, 24]. The 2010 UNAIDS report also suggested that paid sex remains as an important contributor to HIV epidemics in Western, Central, and Eastern Africa [12]. In addition, risky sexual behaviour is strongly associated with better educational attainment [9, 18–20]. HIV infection is also highly prevalent among educated people, even after controlling for age, sex, region, and residence [32].

We suggest that male youth who were rated as middle to high on the wealth index who manifest risky sexual behaviors are at the core of the HIV transmission, although the high burden of sexually transmitted HIV was reported among young women and girls in Southern Sub-Saharan Africa [33, 34]. Another unpublished study by Berhan Y and et al. has also shown that the practice of higher-risk sex among male youth was more than 10-fold higher than female youth. However, we also believe that it would be premature to attempt to implement any prevention strategies that target them, until we know why these youth are practicing risky sex. Of particular concern, several studies have shown that being educated about the consequences of risky sexual practice and cause of HIV transmission does not necessarily result in behavior change [8, 35, 36]. Further, if HIV education and health promotion interventions delivered over the last quarter-century had been successful, the majority of well-educated male youth living in urban areas would not have been practicing higher-risk sex. Our finding that the mean use of condoms during youth's most recent sexual experience in the countries that were included in this analysis was no more than 51% suggests this cohort's continuing resistance to practice unprotected sex. Low rates of condom use among youth have been also reported in other studies [31, 37, 38].

However, UNAIDS reported that the prevalence of HIV among young people has been falling in some of the worst-hit countries around the world coinciding with a change in sexual behaviour patterns [12]. This meta-analysis reveals the high prevalence of risky sexual behaviour in male youth in different parts of the world. The observed behavioral change in the UNAIDS 2010 report must thus be seen in relative terms.

Although a systematic review of evaluations of school-based sexual health interventions suggests improvements in knowledge, attitudes, and intentions, lasting behavioral changes were few [39]. We suggest that the male youth in our sample who were under 20 years of age may have been less likely to have stable sexual partnership than has been previously reported [6, 7, 40]. If so, they are likely to engage in sexual intercourse with women who are neither a spouse nor a cohabiting partner.

This meta-analysis was not without limitation. Since a limited amount of DHS data outside Sub-Saharan Africa was included, it might not be generalizable for all developing countries. Being secondary data analysis limited us from performing analysis for other variables like peer's influence, ethnicity/race, religion, parents relation, and substance use. Furthermore, the dichotomization of the independent variables to fit into the meta-analysis software could not allow us to make comparisons across multiple categories and might have impacts on the degrees of freedom. The fact that the primary studies are cross sectional by design identified only association of sexual behavior with selected variables and helped us to generate hypothesis but was not helpful to identify causality.

In conclusion, consistent with the previous literature [39], we found that male youth aged 15–19 were more likely to engage in higher-risk sexual activity than those aged 20–24 years. In twenty of the countries studied, the highest prevalence of risky sexual behavior occurred among male youth living in urban areas who had completed secondary education and belonged to the middle to the highest wealth index. As previous reviews of SSA countries have demonstrated, there is both a positive-wealth and a positive-education gradient in regard to HIV infection [19, 20, 32]. Although this study supports the relation between education/wealth and higher-risk sex, the mechanisms of this relationship are not clear. More work on the reasons for this link is needed so that prevention activities can be successfully implemented.
